# Perioperative Change of High‐Sensitive Cardiac Troponin I Concentration in Cats According to Three Different Anaesthesia Protocols

**DOI:** 10.1002/vms3.70050

**Published:** 2024-09-25

**Authors:** Tarik Safak, Oznur Yılmaz‐Koç, Ebru Karakaya‐Bilen

**Affiliations:** ^1^ Department of Obstetrics and Gynecology, Faculty of Veterinary Medicine Kastamonu University Kastamonu Türkiye; ^2^ Department of Obstetrics and Gynecology, Faculty of Veterinary Medicine Siirt University Siirt Türkiye; ^3^ Department of Obstetrics and Gynecology, Faculty of Ceyhan Veterinary Medicine Cukurova University Adana Türkiye

**Keywords:** cardiac troponin I, cat, isoflurane, ketamine hydrochloride, propofol, xylazine hydrochloride

## Abstract

**Background:**

Cardiac troponin I, a particular biomarker, is released into the bloodstream in response to myocardial injury.

**Objectives:**

To evaluate perioperative changes in high‐sensitivity cardiac troponin I (hs‐cTnI) concentration during ovariohysterectomy in cats undergoing three different anaesthesia protocols.

**Methods:**

Twenty‐one female mixed‐breed cats owned by clients aged (2.2 ± 0.7 years) and weight (3.2 ± 0.5 kg) were included in our study. The cats were divided into three groups: propofol–isoflurane (PI) group (*n* = 7), xylazine–ketamine (XK) group (*n* = 7) and xylazine–isoflurane (XI) group (*n* = 7). After pre‐anaesthetic propofol (6 mg/kg IV) was administered to cats in Group PI, a mask was placed, and anaesthesia was maintained with 3.0% isoflurane in oxygen. Cats in Group XK underwent general anesthetization with xylazine hydrochloride (2 mg/kg IM) and, 10 min later, ketamine hydrochloride (10 mg/kg IM). Cats in Group XI were administered xylazine hydrochloride (2 mg/kg IM), and then anaesthesia (3.0% isoflurane and oxygen) was continued with a mask. Blood samples were collected from all cats; preoperatively and postoperatively at 0 and 12 h (Pre‐, Post‐0 h and Post‐12 h, respectively). Serum hs‐cTnI concentrations were measured with the Advia Centaur TnI‐Ultra.

**Results:**

In all 21 cats, hs‐cTnI concentration increased at Post‐0 h and 12 h measurement points compared to Pre‐. In the XK group, hs‐cTnI concentrations exhibited a significant increase at the Post‐0 h (51.30 ng/L) and Post‐12 h (157.70 ng/L) time points compared to Pre‐ (6.70 ng/L) (*p* < 0.05).

**Conclusions:**

The XK group increased the concentration of hs‐cTnI more than other protocols. In the PI group, the increase in hs‐cTnI concentrations at Post‐0 and 12 h increased less than the other two groups (*p* < 0.05). The PI group was found to induce less myocardial damage.

## Graphical abstract

• Cardiac troponins have been recognized as a gold standard marker for detecting myocardial injury in humans and animals.

• Perioperative troponin concentration increased more in the xylazine–ketamine protocol than in other anaesthesia protocols.

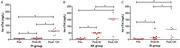

## 1 Introduction

Choosing a safe anaesthesia protocol is a critical component for treating animals during various medical procedures and surgeries in veterinary medicine. Although anaesthetics are crucial in ensuring patient comfort and stability, their impact on the cardiovascular system, specifically myocardial muscle function, remains a subject of significant research and clinical interest (Cilli et al. 2010; Souza et al. 2022; Suarez et al. 2012).

Perioperative myocardial injury is a well‐documented phenomenon associated with anaesthesia, which can lead to detrimental outcomes if not adequately understood and managed. It was recently identified in humans as myocardial damage following non‐cardiac surgery (Puelacher et al. 2018). Myocardial damage during non‐cardiac surgery is observed in 17.9% of humans and is characterized by increased high‐sensitive cardiac troponin I (hs‐cTnI) concentration throughout the perioperative period (Devereaux et al. 2017; Ruetzler et al. 2021). Cardiac troponin I (cTnI), a particular biomarker released into the bloodstream in response to myocardial injury, offers a precise means of evaluating the extent of cardiac compromise during anaesthesia. After general anaesthesia, dogs have reportedly seen a rise in cTnI concentration (Verbiest et al. 2013). On the other hand, the effect of perioperative general anaesthesia protocols on serum cTnI concentration in cats during ovariohysterectomy has not yet been reported. Monitoring serum cTnI concentration during the operation in anesthetized cats undergoing different anaesthesia protocols allows us to assess the degree of myocardial stress and injury induced by these agents.

This study aimed to evaluate perioperative changes in the hs‐cTnI concentration during the ovariohysterectomy in cats. Our investigation measures hs‐cTnI concentration as a critical myocardial injury indicator. To investigate the varied impacts of various anaesthetic protocols, we utilized three distinct protocols: (1) propofol with isoflurane, (2) xylazine with ketamine and (3) xylazine with isoflurane. By comparing the impact of these anaesthesia protocols on serum hs‐cTnI concentration, we aim to uncover valuable insights into the cardiac consequences of anaesthesia administration in cats.

## 2 Materials and Methods

### 2.1 Animals and Study Groups

G‐Power programme was used to determine the number of samples used in the study. In this study, the required minimum sample number was determined as 21 using effect size = 0.40, *α* = 0.05 and power (1 − *β* error level) = 0.90 (Cohen 1988).

Twenty‐one female mixed‐breed cats owned by clients were included in our study. This study was conducted on healthy cats brought to Siirt University's Faculty of Veterinary Medicine Department of Obstetrics and Gynecology for routine elective ovariohysterectomy. The cats had an average age of 2.2 ± 0.7 years and an average body weight of 3.2 ± 0.5 kg. Before the surgery, the cats were assessed as healthy on the basis of their medical history, physical examination, serum biochemical tests and complete blood count. Haematological and biochemical values are within the reference range. Each cat was allocated randomly to one of three treatment groups: propofol–isoflurane (PI) (*n* = 7), xylazine–ketamine (XK) (*n* = 7) and xylazine–isoflurane (XI) (*n* = 7). For preanaesthetic purposes, cats in Group PI administered 6 mg/kg IV propofol (propofol 1% Fresenius, Uppsala, Sweden). Then, a mask was placed on to continue the anaesthetic. Anaesthesia was maintained with 3.0% isoflurane (isoflurane, USP, Bethlehem, Pennsylvania, USA) in oxygen (vaporizer setting, Mindray WATO EX‐20Vet, Mindray Medical International Limited, Shenzhen, China). Cats in Group XK underwent general anaesthesia with xylazine hydrochloride (2 mg/kg IM, Rompun 2%, Bayer, Istanbul, Türkiye) and 10 min later ketamine hydrochloride (10 mg/kg IM, Ketasol 10%, Richter Pharma, Wels, Austria) (Safak and Yilmaz 2023). Cats in Group XI received xylazine hydrochloride (2 mg/kg IM, Rompun 2%, Bayer). The mask was then placed, and the anaesthesia was maintained with 3.0% isoflurane and oxygen (vaporizer setting, Mindray WATO EX‐20Vet, Mindray Medical International Limited).

### 2.2 Surgical Procedures

In all cats, a standard protocol was followed wherein a period of 8 h of food and water restriction was consistently implemented prior to the ovariohysterectomy. The ovariohysterectomy was started after the aseptic surgical standards were prepared. The procedure of ovariohysterectomy was carried out in accordance with the methodology defined by Karakaya‐Bilen et al. (2023). Depending on the operator's preference, queens were positioned in left lateral recumbency when using the lateral flank approach. To prevent variations between surgeries, an ovariohysterectomy was carried out by the same surgeon. Because the same experienced surgeon performed the ovariohysterectomy on each cat, the process took about 10 min per cat. All cats breathed spontaneously during anaesthesia.

### 2.3 Blood Samples and hs‐cTnI Measurement

Blood samples were collected from cats in three groups; the first blood sample was collected during a general examination performed immediately before anaesthesia began (Pre‐). Post‐operative samples were then obtained immediately upon completion of the surgery and 12 h later (Post‐0 h and Post‐12 h, respectively). Blood samples were obtained from the cephalic vein to determine serum hs‐cTnI concentration. These samples were collected into tubes that were without any anticoagulant. The serum was separated by centrifugation at 5000 rpm for 10 min and then stored at −20°C until analysis. After thawing, the serum concentrations of hs‐cTnI in 21 cats were evaluated using a chemiluminescent immunoassay for detecting human‐based hs‐cTnI (ADVIA Centaur XP High‐Sensitivity Troponin I, Siemens Healthcare Diagnostics). According to the manufacturer, the measurement range of this assay is 2.5–25,000 ng/L. This human‐based analyser has been validated in dogs (Wesselowski et al. 2023) but has not yet been validated in cats. The previous generation of this assay, the ADVIA Centaur CP TnI‐Ultra, was validated in both dogs (Winter et al. 2014) and cats (Langhorn et al. 2013) but is no longer commercially available.

### 2.4 Statistical Analyses

The normality distribution of the hs‐cTnI was tested using the visual (histogram and probability graphs) and Shapiro–Wilk test. On the basis of the assessment, it was concluded that there was an absence of a normal distribution of characteristic values within each group. Therefore, the Kruskal–Wallis test, a non‐parametric statistical technique commonly employed for comparing different groups, was used for inter‐group comparisons. The findings are displayed as median (min–max). The post hoc pairwise group comparisons following the Kruskal–Wallis test were conducted using the Bonferroni‐corrected Mann–Whitney *U*‐test. The Friedman test for repeated measurements was used to compare non‐parametric data among three measurement points. Wilcoxon test was then applied for post hoc analysis. SPSS 22 (Statistical Package for the Social Sciences, Chicago, USA) was applied for the statistical analyses.

## 3 Results

On the basis of the anaesthetic groups’ (PI, XK and XI) investigation findings, the XK group exhibited a significant increase in hs‐cTnI concentration at the Post‐0 h (51.30 ng/L) (*p* = 0.021) time point. The observed rise persisted over the Post‐12 h period, with a recorded value of 157.70 ng/L, which remained significantly elevated compared to the other groups (*p* = 0.038). There was no statistically significant difference between the groups’ preoperative concentrations of hs‐cTnI (*p* = 0.158) (Table 1).

**TABLE 1:** Median (minimum–maximum) of high‐sensitive cardiac troponin I concentrations at different times in the three groups.

Groups	Time points			*p*
	Pre‐ (*n* = 7)	Post‐0 h (*n* = 7)	Post‐12 h (*n* = 7)	
	Median (ng/L) (min–max)	Median (ng/L) (min–max)	Median (ng/L) (min–max)	
PI	7.40 (5.40–8.40)^a^	8.60 (6.10–18.30)^Ab^	61.40 (29.90–84.10)^Ac^	0.001
XK	6.70 (3.40–15.90)^a^	51.30 (8.80–51.80)^Bb^	157.70 (24.50–206.10)^Bc^	0.000
XI	4.80 (2.50–11.40)^a^	15.50 (3.50–84.60)^ABb^	77.30 (12.00–234.70)^ABc^	0.002
*p*	0.158	0.021	0.038	

*Note*: The difference between groups with different letters (A, B) in the same column is statistically significant (*p* < 0.05). The difference between measurement points with different letters (a, b, c) on the same line is statistically significant (*p* < 0.05).

Abbreviations: PI, propofol–isoflurane; XI, xylazine–isoflurane; XK, xylazine–ketamine.

**FIGURE 1:**
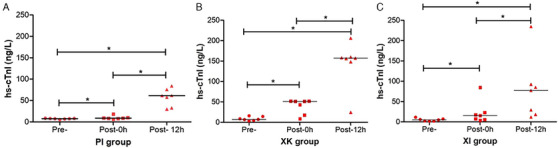
High‐sensitive cardiac troponin I (hs‐cTnI) concentrations at three measurement points (preoperation, postoperation 0 h and 12 h) of the PI (A), XK (B) and XI (C) groups. PI, propofol–isoflurane; XI, xylazine–isoflurane; XK, xylazine–ketamine. Asterisks indicate statistical difference (*p < 0.05).

In all 21 cats (100%), hs‐cTnI concentration increased at Post‐0 h and 12 h measurement points compared to Pre‐. According to the hs‐cTnI measurement points of PI, XK and XI groups, *p* values were calculated as 0.001, 0.000 and 0.002, respectively. All three anaesthesia protocols exhibited an increase in hs‐cTnI concentration as measured at Post‐0 h points after the operation (*p* < 0.001). In addition, an upsurge was detected in the hs‐cTnI measurement performed at Post‐12 h compared to Post‐0 h (*p* < 0.001) (Table 1, Figure 1).

## 4 Discussion

The present investigation aimed to examine the influence of different anaesthesia protocols on perioperative serum hs‐cTnI concentration in cats, focusing on assessing potential variations in myocardial injury associated with anaesthesia. This study found that all three anaesthesia protocols had higher postoperative serum hs‐cTnI concentrations. The XK group experienced a more significant increase than the other two groups.

Cardiac troponins have been recognized as a gold standard marker for detecting myocardial injury in humans (Sarko and Pollack 2002; Wu and Jaffe 2008) and animals (Karapinar et al. 2012; Langhorn and Willesen 2016; Leonardi et al. 2008; Tumer and Safak 2022). The previous research has demonstrated that the concentration of circulating cTnI rises in various animals (Tümer, Çalışkan, and Safak 2021) and experimental disease models (Diniz et al. 2008; Tümer, Özdemir, and Eröksüz 2020a; Tümer, Özdemir, and Eröksüz 2020b). cTnI has been reported to be elevated in cats and dogs with or without cardiac systemic disease (Porciello et al. 2008). Serum cTnI concentrations in anaemic cats were greater than in non‐anaemic sick cats (Lalor et al. 2014). In a study conducted on canines with babesiosis, increased serum cTnI concentrations were reported (Lobetti, Dvir, and Pearson 2008). Another study on canines with babesiosis, a high concentration of cTnI was found to correlate with both the severity of the disease and a poor prognosis (Lobetti, Reyers, and Nesbit 1996). Anaesthesia is administered to animals for various purposes, similar to its application in humans. Research is now being conducted to determine the relative safety of different anaesthetic drugs (Suarez et al. 2012). The heart is one of the organs most impacted by anaesthesia. As a result, research is being conducted to uncover the detrimental consequences on the heart. cTnI concentration determination is currently one of the most accurate approaches for assessing myocardial injury throughout anaesthesia (Wolfe Barry, Barth, and Howell 2008). Although perioperative research has been carried out on dogs (Verbiest et al. 2013), there are not enough studies on cats. The effect of XK and PI combinations, which are commonly utilized protocols today, on serum hs‐cTnI concentration was investigated in this study. In a study conducted by Tocheto et al. (2015) on cats, cats administered a combination of ketamine and midazolam exhibited an increase in cTnI concentration after surgery. Even in the absence of a surgery, dexmedetomidine administration caused changes in echocardiographic results and circulating hs‐cTnI in cats. They demonstrated substantial increases in cTnI in cats after administration of dexmedetomidine (Côté et al. 2022). Alpha‐2 adrenergic receptor agonists reliably and dose‐dependently induce sedation, analgesia and muscle relaxation in dogs and cats (Maze and Tranquilli 1991). These effects can be easily reversed by administering selective antagonists (yohimbine, atipamezole). Xylazine and dexmedetomidine are both approved for use as alpha‐2 agonists in small animals (Bloor et al. 1992; Lemke 2004). Alpha‐2 agonists initially induce constriction of blood vessels in the periphery, leading to a temporary increase in blood pressure, a decrease in heart rate and a reduction of up to 50% in the amount of blood pumped by the heart. Subsequently, the alpha‐2 agonists induce a sympatholytic effect, resulting in the expansion of peripheral blood vessels and a decrease in heart rate (Granholm et al. 2006; McSweeney et al. 2012; Willey et al. 2016). Although subclinical myocardial injury may go unnoticed in many cats, cardiac necrosis and peri‐anaesthetic death are reported in some cats, particularly in those with alpha‐2 agonist agents used in their anaesthetic protocols (Van der Linde‐Sipman, Hellebrekers, and Lagerwey 1992). In our study, no cat died after anaesthesia and surgery. However, both the current study and previous research indicate that alpha‐2 agonist may be the cause of subclinical myocardial injury in cats. It is important to be more careful when using anaesthetic agents. These data suggest that more investigation into myocardial injury in cats that receive alpha‐2 agonist is warranted.

In previous studies conducted on dogs, Saunders et al. (2009) reported that the perioperative cTnI concentration did not change in the propofol and diazepam groups, which are two different anaesthesia protocols. In a study conducted by Cilli et al. (2010), it was observed that concentrations of cTnI exhibited a rise following anaesthesia in only 21 out of 102 dogs. Anaesthesia with propofol, with or without premedication with medetomidine, has been linked to increased serum cTnI concentrations that cause subclinical myocardial injury (Vasiljević et al. 2018). It demonstrates that using atropine before anaesthesia with 5 g/kg dexmedetomidine does not affect the cTnI level. However, it was discovered that combining atropine with 10 g/kg dexmedetomidine caused subclinical myocardial injury with increasing cTnI concentration (Huang et al. 2021).

Although various anaesthetic protocols have different effects on the concentration of cTnI, the animal's age also impacts the concentration of cTnI (Winter et al. 2014; Wesselowski et al. 2023). Even in the absence of significant heart disease, cTnI concentration has been found to correlate with the age of dogs (Oyama and Sisson 2004). When compared to younger, older dogs (>8 years) had a 3.6‐times higher increase in plasma cTnI concentration (Cilli et al. 2010). In this study, however, cats under 3 years of age were enrolled to exclude age‐related variables. Meanwhile, as the duration of the surgical procedure extends, there is a corresponding increase in the concentration of cTnI, primarily attributed to prolonged exposure to anaesthetic (Verbiest et al. 2013). In this study, an ovariohysterectomy was conducted, and the duration of the operation for each cat was approximately 10 min.

Konishi et al. (2022) found that after surgery, females had higher hs‐cTnI levels than males. This result is not unexpected given that castration is less invasive and less time‐consuming than ovariohysterectomy. In the same study, hs‐cTnI concentrations were considerably greater Post‐0 h and 18 h than preoperatively. Our results, like Konishi et al. (2022), showed that the concentration of cTnI in the serum at 0 and 12 h after surgery was higher than before. The outcome of this study is in‐line with expectations. Because the cats used in the study were selected to ensure homogeneity and were randomly assigned to one of three groups.

Troponin's stability is preserved between −70°C and −80°C for 12–24 months (Basit et al. 2007; Langhorn and Willesen 2016). On the other hand, it has been reported to be kept at 4°C for up to 2 weeks. At −20°C, it is not recommended for long‐term storage. At −20°C, short‐term storage is possible for up to 3 months (Langhorn and Willesen 2016; Woltersdorf, Bayly, and Day 2001). According to these findings, the serum was promptly stored in the freezer at a temperature of −20°C after collection. Because of this, hs‐cTnI analyses were conducted within a 30‐day period in our study.

This study had some limitations in its design. First, the ability to identify risk variables for elevated hs‐cTnI concentrations following anaesthesia is limited due to the relatively small number of animals in the groups. Second, although the concentration of hs‐cTnI varies depending on the severity of myocardial injury, it usually begins to rise within 6 h following the damage. It has been reported that this rise reaches its peak within 12–24 h, lasting up to 2 weeks (Nigam 2007; Wolfe Barry, Barth, and Howell 2008). In this study, hs‐cTnI could not be tested longer because the cats were only in the hospital for a short time and the owners would not let them stay longer. When the cats were checked out a week after surgery, they had no signs of heart problems. It is still unclear whether higher hs‐cTnI in cats is reversible. Unfortunately, this study could not determine whether or not this increase was reversible. Another limitation is that cats were not subjected to an echocardiogram prior to anaesthesia and ovariohysterectomy; thus, the presence of underlying cardiac disease cannot be excluded as a confounding factor in hs‐cTnI elevations after anaesthesia.

## 5 Conclusion

After the operation, the concentration of hs‐cTnI in all cats was elevated compared to preoperation. The combination of XK increased the concentration of hs‐cTnI more than other protocols. The combination of PI was found to induce less myocardial damage. The measurement of hs‐cTnI appears to be beneficial in detecting perioperative myocardial injury. Moreover, to better understand the change in perioperative hs‐cTnI, postoperative observation is required for a more extended period, that is, hs‐cTnI measurement.

## Author Contributions

**Tarik Safak**: investigation, resources, writing manuscript draft, review and editing. **Oznur Yilmaz‐Koç** and **Ebru Karakaya‐Bilen**: collected the blood samples, laboratory activities, review and editing. All authors read and approved the final manuscript.

## Ethics Statement

The study was approved by the Local Ethical Committee of Siirt University (2022/02/08).

## Consent

The owners of the cats approved the informed consent form.

## Conflicts of Interest

The authors declare no conflicts of interest.

## Peer Review

The peer review history for this article is available at https://publons.com/publon/10.1002/vms3.70050.

## Data Availability

The data that support the findings of this study are available from the corresponding author, upon reasonable request.

## References

[vms370050-bib-0001] Basit, M. , N. Bakshi , M. Hashem , et al. 2007. “The Effect of Freezing and Long‐Term Storage on the Stability of Cardiac Troponin T.” American Journal of Clinical Pathology 128, no. 1: 164–167. 10.1309/LR7FC0LUGLHT8X6J. 17580285

[vms370050-bib-0002] Bloor, B. C. , M. Frankland , G. Alper , D. Raybould , J. Weitz , and M. Shurtliff . 1992. “Hemodynamic and Sedative Effects of Dexmedetomidine in Dog.” Journal of Pharmacology and Experimental Therapeutics 263, no. 2: 690–697. 1359110

[vms370050-bib-0003] Cilli, F. , H. I. Alibhai , E. Armitage‐Chan , et al. 2010. “Incidence of Elevation of Cardiac Troponin I Prior to and Following Routine General Anaesthesia in Dogs.” Veterinary Anaesthesia and Analgesia 37, no. 5: 409–416. 10.1111/j.1467-2995.2010.00554.x. 20712607

[vms370050-bib-0004] Cohen, J. 1988. Statistical Power Analysis for the Behavioral Sciences. Mahwah, NJ: Lawrence.

[vms370050-bib-0005] Côté, E. , L. A. Zwicker , E. L. Anderson , H. Stryhn , J. Yu , and E. Andersen . 2022. “Effects of Dexmedetomidine and Its Reversal With Atipamezole on Echocardiographic Measurements and Circulating Cardiac Biomarker Concentrations in Normal Cats.” Journal of the American Veterinary Medical Association 260, no. 8: 1–9. 10.2460/javma.21.06.0299. 35175929

[vms370050-bib-0006] Devereaux, P. J. , B. M. Biccard , A. Sigamani , D. Xavier , M. T. Chan , and S. K. Srinathan . 2017. “Association of Postoperative High‐Sensitivity Troponin Levels With Myocardial Injury and 30‐Day Mortality Among Patients Undergoing Noncardiac Surgery.” Journal of the American Medical Association 317, no. 16: 1642–1651. 10.1001/jama.2017.4360. 28444280

[vms370050-bib-0007] Diniz, P. P. V. P. , H. S. A. De Morais , E. B. Breitschwerdt , and D. S. Schwartz . 2008. “Serum Cardiac Troponin I Concentration in Dogs With Ehrlichiosis.” Journal of Veterinary Internal Medicine 22, no. 5: 1136–1143. 10.1111/j.1939-1676.2008.0145.x. 18638021

[vms370050-bib-0008] Granholm, M. , B. C. McKusick , F. C. Westerholm , and J. C. Aspegrén . 2006. “Evaluation of the Clinical Efficacy and Safety of Dexmedetomidine or Medetomidine in Cats and Their Reversal With Atipamezole.” Veterinary Anaesthesia and Analgesia 33, no. 4: 214–223. 10.1111/j.1467-2995.2005.00259.x. 16764585

[vms370050-bib-0009] Huang, H. Y. , K. Y. Liao , W. Y. Shia , C. C. Chang , and H. C. Wang . 2021. “Effect of Administering Dexmedetomidine With or Without Atropine on Cardiac Troponin I Level in Isoflurane‐Anesthetized Dogs.” Journal of Veterinary Medical Science 83, no. 12: 1869–1876. 10.1292/jvms.20-0657. 34629333 PMC8762405

[vms370050-bib-0010] Karakaya‐Bilen, E. , S. K. Demirbilek , Ö. Yılmaz , M. A. Karadağ , and H. Aner . 2023. “Comparison of Bacterial Profile and Antibiotic Susceptibility Isolated From Surgical Site After Ventral Midline and Lateral Flank Approaches of Ovariohysterectomy in Queens and Bitches.” Turkish Journal of Veterinary & Animal Sciences 47, no. 4: 316–323. 10.55730/1300-0128.4300.

[vms370050-bib-0011] Karapinar, T. , Y. Eroksuz , E. Beytut , I. Sozdutmaz , H. Eroksuz , and M. Dabak . 2012. “Increased Plasma Cardiac Troponin I Concentration in Lambs With Myocarditis.” Veterinary Clinical Pathology 41, no. 3: 375–381. 10.1111/j.1939-165X.2012.00448.x. 22747688

[vms370050-bib-0012] Konishi, K. , M. Sakamoto , C. Satake , et al. 2022. “Perioperative Changes in Cardiac Biomarkers in Juvenile Cats During Neutering.” Frontiers in Veterinary Science 9: 1008765. 10.3389/fvets.2022.1008765. 36268044 PMC9577090

[vms370050-bib-0013] Lalor, S. M. , D. A. Gunn‐Moore , R. Cash , A. Foot , N. Reed , and R. J. Mellanby . 2014. “Serum Cardiac Troponin I Concentrations in Cats With Anaemia – A Preliminary, Single‐Centre Observational Study.” Journal of Small Animal Practice 55, no. 6: 320–322. 10.1111/jsap.12210. 24645736

[vms370050-bib-0014] Langhorn, R. , and J. L. Willesen . 2016. “Cardiac Troponins in Dogs and Cats.” Journal of Veterinary Internal Medicine 30, no. 1: 36–50. 10.1111/jvim.13801. 26681537 PMC4913658

[vms370050-bib-0015] Langhorn, R. , J. L. Willesen , I. Tarnow , and M. Kjelgaard‐Hansen . 2013. “Evaluation of a High‐Sensitivity Assay for Measurement of Canine and Feline Serum Cardiac Troponin I.” Veterinary Clinical Pathology 42, no. 4: 490–498. 10.1111/vcp.12085. 24131244

[vms370050-bib-0016] Lemke, K. A. 2004. “Perioperative Use of Selective Alpha‐2 Agonists and Antagonists in Small Animals.” Canadian Veterinary Journal 45, no. 6: 475–480. PMC548630 15283516

[vms370050-bib-0017] Leonardi, F. , B. Passeri , A. Fusari , et al. 2008. “Cardiac Troponin I (cTnI) Concentration in an Ovine Model of Myocardial Ischemia.” Research in Veterinary Science 85, no. 1: 141–144. 10.1016/j.rvsc.2007.09.010. 17961616

[vms370050-bib-0018] Lobetti, R. , E. Dvir , and J. Pearson . 2008. “Cardiac Troponins in Canine Babesiosis.” Journal of Veterinary Internal Medicine 16, no. 1: 63–68. 10.1111/j.1939-1676.2002.tb01607.x. 11822806

[vms370050-bib-0019] Lobetti, R. , F. Reyers , and J. W. Nesbit . 1996. “The Comparative Role of Haemoglobinaemia and Hypoxia in the Development of Canine Babesial Nephropathy.” Journal of the South African Veterinary Association 67, no. 4: 188–198. 9284030

[vms370050-bib-0020] Maze, M. , and W. Tranquilli . 1991. “Alpha‐2 Adrenoceptor Agonists: Defining the Role in Clinical Anesthesia.” Anesthesiology 74: 581–605. 10.1097/00000542-199103000-00029. 1672060

[vms370050-bib-0021] McSweeney, P. M. , D. D. Martin , D. S. Ramsey , and B. C. McKusick . 2012. “Clinical Efficacy and Safety of Dexmedetomidine Used as a Preanesthetic Prior to General Anesthesia in Cats.” Journal of the American Veterinary Medical Association 240, no. 4: 404–412. 10.2460/javma.240.4.404. 22309012

[vms370050-bib-0022] Nigam, P. K. 2007. “Biochemical Markers of Myocardial Injury.” Indian Journal of Clinical Biochemistry 22, no. 1: 10–17. 10.1007/BF02912874. 23105645 PMC3454263

[vms370050-bib-0023] Oyama, M. A. , and D. D. Sisson . 2004. “Cardiac Troponin‐I Concentration in Dogs With Cardiac Disease.” Journal of Veterinary Internal Medicine 18, no. 6: 831–839. 10.1111/j.1939-1676.2004.tb02629.x. 15638266

[vms370050-bib-0024] Porciello, F. , M. Rishniw , W. E. Herndon , F. Birettoni , M. T. Antognoni , and K. W. Simpson . 2008. “Cardiac Troponin I Is Elevated in Dogs and Cats With Azotaemia Renal Failure and in Dogs With Non‐Cardiac Systemic Disease.” Australian Veterinary Journal 86, no. 10: 390–394. 10.1111/j.1751-0813.2008.00345.x. 18826510

[vms370050-bib-0025] Puelacher, C. , G. Lurati Buse , D. Seeberger , et al. 2018. “Perioperative Myocardial Injury After Noncardiac Surgery: Incidence, Mortality, and Characterization.” Circulation 137, no. 12: 1221–1232. 10.1161/CIRCULATIONAHA.117.030114. 29203498

[vms370050-bib-0026] Ruetzler, K. , N. R. Smilowitz , J. S. Berger , et al. 2021. “Diagnosis and Management of Patients With Myocardial Injury After Noncardiac Surgery: A Scientific Statement From the American Heart Association.” Circulation 144, no. 19: 287–305. 10.1161/CIR.0000000000001024. 34601955

[vms370050-bib-0027] Safak, T. , and O. Yilmaz . 2023. “Bioquímica sérica y perfil hematológico de una gata con tres fetos momificados.” Journal MVZ Córdoba 28, no. 1: 1–5. 10.21897/rmvz.2778.

[vms370050-bib-0028] Sarko, J. , and C. V. Pollack . 2002. “Cardiac Troponins.” Journal of Emergency Medicine 23, no. 1: 57–65. 10.1016/S0736-4679(02)00463-8. 12217473

[vms370050-bib-0029] Saunders, A. B. , A. S. Hanzlicek , E. A. Martinez , et al. 2009. “Assessment of Cardiac Troponin I and C‐Reactive Protein Concentrations Associated With Anesthetic Protocols Using Sevoflurane or a Combination of Fentanyl, Midazolam, and Sevoflurane in Dogs.” Veterinary Anaesthesia and Analgesia 36, no. 5: 449–456. 10.1111/j.1467-2995.2009.00483.x. 19709049

[vms370050-bib-0030] Souza, T. , N. dos Anjos , I. Kersul , E. Martins Filho , T. Nunes , and V. Barbosa . 2022. “Effects of Dexmedetomidine or Tramadol Continuous Rate Infusions on the Propofol Requirements and Cardiorespiratory Variables in Propofol‐Ketamine‐Midazolam Anaesthetised Cats.” Veterinární Medicína 67, no. 4: 199–205. 10.17221/138/2020-VETMED. 39170806 PMC11334771

[vms370050-bib-0031] Suarez, M. A. , B. T. Dzikiti , F. G. Stegmann , and M. Hartman . 2012. “Comparison of Alfaxalone and Propofol Administered as Total Intravenous Anaesthesia for Ovariohysterectomy in Dogs.” Veterinary Anaesthesia and Analgesia 39, no. 3: 236–244. 10.1111/j.1467-2995.2011.00700.x. 22405473

[vms370050-bib-0032] Tocheto, R. , V. S. Padilha , H. M. Cardoso , et al. 2015. “Troponin I, Electrocardiography and Echocardiography Values in Felines Sedated With Ketamine and Midazolam, Supplemented or Not With Oxygen.” Arquivo Brasileiro De Medicina Veterinária E Zootecnia 67, no. 6: 1572–1580. 10.1590/1678-4162-8136.

[vms370050-bib-0033] Tumer, K. C. , and T. Safak . 2022. “Serum Cardiac Troponin I Concentration Increases in Sheep With Uterine Torsion.” Small Ruminant Research 216: 106784. 10.1016/j.smallrumres.2022.106784.

[vms370050-bib-0034] Tümer, K. Ç. , H. Özdemir , and H. Eröksüz . 2020b. “Investigation of the Myocardial Damage due to Acute Anemia in a Rabbit Model of Acute Normovolemic Anemia.” Turkish Journal of Veterinary & Animal Science 44: 600–606. 10.3906/vet-2001-96.

[vms370050-bib-0035] Tümer, K. Ç. , H. Özdemir , and H. Eröksüz . 2020a. “Evaluation of Cardiac Troponin I in Serum and Myocardium of Rabbits With Experimentally Induced Polymicrobial Sepsis.” Experimental Animals 69, no. 1: 54–61. 10.1538/expanim.19-0046. 31462610 PMC7004812

[vms370050-bib-0036] Tümer, K. Ç. , M. Çalışkan , and T. Safak . 2021. “Serum Cardiac Troponin I Concentrations in Ewes Diagnosed With Parturient Paresis Correlation With Blood Ionized Calcium and Conventional Cardiac Enzymes.” Large Animal Reviver 27: 143–147.

[vms370050-bib-0037] Van der Linde‐Sipman, J. S. , L. J. Hellebrekers , and E. Lagerwey . 1992. “Myocardial Damage in Cats That Died After Anaesthesia.” Veterinary Quarterly 14, no. 3: 91–94. 10.1080/01652176.1992.9694340. 1413446

[vms370050-bib-0038] Vasiljević, M. , V. Krstić , S. Stanković , P. Zrimšek , A. N. Svete , and A. Seliškar . 2018. “Cardiac Troponin I in Dogs Anaesthetized With Propofol and Sevoflurane: The Influence of Medetomidine Premedication and Inspired Oxygen Fraction.” Veterinary Anaesthesia and Analgesia 45, no. 6: 745–753. 10.1016/j.vaa.2018.07.003. 30309716

[vms370050-bib-0039] Verbiest, T. , D. Binst , T. Waelbers , E. Coppieters , and I. Polis . 2013. “Perioperative Changes in Cardiac Troponin I Concentrations in Dogs.” Research in Veterinary Science 94, no. 3: 446–448. 10.1016/j.rvsc.2012.10.023. 23178045

[vms370050-bib-0040] Wesselowski, S. , J. Lidbury , A. B. Saunders , S. G. Gordon , J. S. Suchodolski , and J. M. Steiner . 2023. “Analytical Validation, Sample Stability, and Clinical Evaluation of a New High‐Sensitivity Cardiac Troponin I Immunoassay for Use in Dogs, With Comparison to a Previous Ultrasensitive Assay.” PLoS ONE 18, no. 7: e0288801. 10.1371/journal.pone.0288801. 37463140 PMC10353792

[vms370050-bib-0041] Willey, J. L. , T. M. Julius , S. P. A. Claypool , and M. C. Clare . 2016. “Evaluation and Comparison of Xylazine Hydrochloride and Dexmedetomidine Hydrochloride for the Induction of Emesis in Cats: 47 Cases (2007–2013).” Journal of the American Veterinary Medical Association 248, no. 8: 923–928. 10.2460/javma.248.8.923. 27031419

[vms370050-bib-0042] Winter, R. L. , A. B. Saunders , S. G. Gordon , et al. 2014. “Analytical Validation and Clinical Evaluation of a Commercially Available High‐Sensitivity Immunoassay for the Measurement of Troponin I in Humans for Use in Dogs.” Journal of Veterinary Cardiology 16, no. 2: 81–89. 10.1016/j.jvc.2014.03.002. 24834862

[vms370050-bib-0043] Wolfe Barry, J. A. , J. H. Barth , and S. J. Howell . 2008. “Cardiac Troponins: Their Use and Relevance in Anaesthesia and Critical Care Medicine.” Anaesthesia Critical Care and Pain 8, no. 2: 62–66. 10.1093/bjaceaccp/mkn001.

[vms370050-bib-0044] Woltersdorf, W. W. , G. R. Bayly , and A. P. Day . 2001. “Practical Implications of In Vitro Stability of Cardiac Markers.” Annals of Clinical Biochemistry 38, no. 1: 61–63. 10.1258/0004563011900146. 11270845

[vms370050-bib-0045] Wu, A. H. B. , and A. S. Jaffe . 2008. “The Clinical Need for High‐Sensitivity Cardiac Troponin Assays for Acute Coronary Syndromes and the Role for Serial Testing.” American Heart Journal 155, no. 2: 208–214. 10.1016/j.ahj.2007.10.016. 18215588

